# Safe involvement of first year residents in patients care

**DOI:** 10.4103/0974-2700.44688

**Published:** 2009

**Authors:** Hanan M F Al Kadri

**Affiliations:** Department of Obstetrics and Gynecology, King Saud Bin Abdulaziz University for Health Sciences, College of Medicine, King Abdulaziz Medical City, Riyadh, Saudi Arabia

Sir,

Learners are more likely to benefit from tasks and activities that they can successfully accomplish with the assistance and support of more competent individuals. This assistance can take variety of forms that first year residents are at a maximum need.[1] These junior residents lack the clinical experience, knowledge, and familiarity with the system at their working place. Hence, they are more vulnerable to medical errors resulting from lack of various models of needed senior assistance. This vulnerability will be even more when they work in specialties where acute live saving emergencies are likely to happen.

Students in the clinical areas are frequently thrown into unplanned activities with patients.[2] All of us can recall many cases where junior residents were left alone to do certain tasks due, for example, to shortage of staff. The resulting anxiety and feeling of vulnerability of the residents is a matter of concern.[2] Residents are sometimes confused when it comes to differentiating between their roles as learners and their roles as workers. Highly structured words with rigid task allocation and where strict hierarchical system exists, are unlikely to meet the learning needs of the students.[3] Hence, strong relationship between the residents, their instructors and other health care providers is necessary to break the hierarchical system barriers between them and enhance learning through role model.

The residents should be given the opportunity to observe, practice and reflect on what they see or what is done by their attached seniors. They should be given enough time to do so prior to involving in patients care. Furthermore, to guarantee a safe clinical practice, we should always try to make sure that the new residents acquire the required skills and theoretical knowledge.[4] Health education planner should find a way to boost the residents' skills and knowledge prior to starting the actual clinical work. More importantly, setting a clear role to the residents in the clinical management will enhance patients' safety and positive learning environment.[5]

Increasing the number of sessions and supervised small group discussions at the beginning of the training may lead to positive collaboration and task distribution. Many residents may speak more openly when their audience is smaller. This may indicate the need for more preparatory sessions for the newly starting residents prior to their involvement in patients' care particularly high risk.

Though residents should not be left working independently at the beginning of their training and should be properly guided by their seniors,[4] overdoing this might lead the residents feel less inclined to explore, and might foster dependence rather than self regulated learning. Hence a balance is required.

Finally we can say, improving the clinical teaching and clinical environment should be a major aim of any medical educational program planner. The most important improvement action is to dig deep looking for factors leading to new residents' satisfaction within the clinical learning environment while maintaining high standard of patients' safety. Such knowledge is important particularly when dealing with specialities where stressful acute emergencies are commonly faced.
